# How strong is the rhythm of perception? A registered replication of Hickok *et al*. (2015)

**DOI:** 10.1098/rsos.220497

**Published:** 2025-06-11

**Authors:** Molly J. Henry, Jonas Obleser, Maria R. Crusey, Emily R. Fuller, Yune Sang Lee, Martin Meyer, Elizabeth A. M. Acosta, Stephen C. Van Hedger, Maya Inbar, Chantal Oderbolz, Sienna A. Dunham, Yathida Anankul, Lauren E. Sabo, Christian Keitel, Ross K. Maddox, Kendra Mehl, Gizem Aslan, Peter A. Martens, Sebastian Sauppe, Meir Horovitz, Elizabeth E. Kinghorn, Stratos Koukouvinis, Hans Rutger Bosker, Mert Huviyetli, Carole Leung, Ashley Elizabeth Symons, Antje Strauß, Maria Chait, Mingyue Hu, Carsten Eulitz, Cailey A. Salagovic, Chris Davis, Giulio Glauco Adriaan Severijnen, Alexandra I. Kosachenko, Claude Alain, Jeesun Kim, Jessica A. Grahn, Riya K. Sidhu, Carlo Megighian, Blake E. Butler, David R. W. Sears, Björn Herrmann, Megan Louise Griffiths, Ayelet N. Landau, Raha Razin, Massimo Grassi, Andrew Levitsky, Lori L. Holt, Amy M. Belfi, Hannah J. Stewart, Barbara G. Shinn-Cunningham, Christi Gomez, Faye Brookes, Erin D. Smith, Ethan Axler, Karin Bakardjian, Daniel Hochstrasser, Lucrezia Guiotto Nai Fovino, Sarah Tune, Yuri G. Pavlov, Kalysta A. Lee, Ashlynne G. Xavier, Anne Keitel, Chad S. Rogers, Ann Maltseva, Julia L. Strauss, Facundo F. Lodol, Naeem Arsiwala, Jonathan E. Peelle

**Affiliations:** ^1^ Max Planck Institute for Empirical Aesthetics, Frankfurt am Main, Germany; ^2^ Department of Psychology, University of Lübeck, Lubeck, Schleswig-Holstein, Germany; ^3^ Center of Brain, Behaviour, and Metabolism, University of Lübeck, Lubeck, Schleswig-Holstein, Germany; ^4^ Department of Otolaryngology, Washington University in St Louis, St Louis, MO, USA; ^5^ Department of Psychological Science, Missouri University of Science and Technology, Rolla, MO, USA; ^6^ School of Behavioral and Brain Sciences, The University of Texas at Dallas, Richardson, TX, USA; ^7^ Institute for the Interdisciplinary Study of Language Evolution, University of Zurich, Zürich, Switzerland; ^8^ Department of Interdisciplinary Arts, Texas Tech University, Lubbock, TX, USA; ^9^ Department of Psychology, Huron University, London, Ontario, Canada; ^10^ Department of Linguistics, The Hebrew University of Jerusalem, Jerusalem, Israel; ^11^ Department of Psychology, The Hebrew University of Jerusalem, Jerusalem, Israel; ^12^ Department of Cognitive and Brain Sciences, The Hebrew University of Jerusalem, Jerusalem, Israel; ^13^ Department of Neuroscience, Georgetown University Medical Center, Washington, DC, USA; ^14^ Department of Biology, Union College, Schenectady, NY, USA; ^15^ Department of Neuroscience, University of Rochester, Rochester, NY, USA; ^16^ Department of Biomedical Engineering, University of Rochester, Rochester, NY, USA; ^17^ Neuroscience Institute, Carnegie Mellon University, Pittsburgh, PA, USA; ^18^ Department of Psychology, University of Stirling, Stirling, UK; ^19^ Department of Psychology, University of Dundee, Dundee, UK; ^20^ Department of Rehabilitation Sciences, Ghent University, Ghent, Flanders, Belgium; ^21^ Department of Psychology, University of Zurich, Zürich, Zürich, Switzerland; ^22^ Donders Institute for Brain, Cognition, and Behaviour, Radboud University Nijmegen, Nijmegen, Gelderland, The Netherlands; ^23^ University College London Ear Institute, London, UK; ^24^ Faculty of Health Sciences, Izmir Bakircay University, Izmir, Turkiye; ^25^ Department of Psychology, Royal Holloway University of London, Egham, UK; ^26^ Department of Psychological Sciences, Birkbeck, University of London, London, UK; ^27^ Department of Linguistics, University of Konstanz, Konstanz, Baden-Württemberg, Germany; ^28^ Department of Psychology, Western University, London, Ontario, Canada; ^29^ The MARCS Institute for Brain, Behaviour, and Development, Western Sydney University, Penrith, New South Wales, Australia; ^30^ Laboratory of neurotechnology, Ural Federal University, Yekaterinburg, Sverdlovsk Oblast, Russian Federation; ^31^ Rotman Research Institute, Baycrest Academy for Research and Education, Toronto, Ontario, Canada; ^32^ Department of General Psychology, University of Padua, Padua, Veneto, Italy; ^33^ Department of Psychology, Lancaster University, Lancaster, UK; ^34^ Department of Experimental Psychology, University College London, London, UK; ^35^ Division of Psychology and Language Sciences, University College London, London, UK; ^36^ Lab in Multisensory Neuroscience, Carnegie Mellon University, Pittsburgh, PA, USA; ^37^ Department of Psychology, The University of Texas at Austin, Austin, TX, USA; ^38^ Department of Psychology, Missouri University of Science and Technology, Rolla, MO, USA; ^39^ Department of Psychology, Carnegie Mellon University, Pittsburgh, PA, USA; ^40^ Department of Speech and Hearing Sciences, University of Washington, Seattle, WA, USA; ^41^ Institute of Medical Psychology and Behavioral Neurobiology, University of Tübingen, Tübingen, Baden-Württemberg, Germany; ^42^ Department of Psychology, Union College, Schenectady, NY, USA; ^43^ Ural Federal University, Yekaterinburg, Sverdlovsk Oblast, Russian Federation; ^44^ Center for Cognitive and Brain Health, Northeastern University, Boston, MA, USA; ^45^ Department of Communication Sciences and Disorders, Northeastern University, Boston, MA, USA; ^46^ Department of Psychology, Northeastern University, Boston, MA, USA

**Keywords:** auditory perception, rhythm perception, entrainment

## Abstract

Our ability to predict upcoming events is a fundamental component of human cognition. One way in which we do so is by exploiting temporal regularities in sensory signals: the ticking of a clock, falling of footsteps and the motion of waves each provide a structure that may facilitate anticipation. But how strong is the effect of rhythmic anticipation on perception? And to what degree do people vary in their ability to capitalize on these regularities? In 2015, Hickok *et al*. introduced a behavioural paradigm to assess how a rhythmic auditory stimulus affects perception of subsequent targets (Hickok G, Farahbod H, Saberi K. 2015 The rhythm of perception: entrainment to acoustic rhythms induces subsequent perceptual oscillation. *Psychol. Sci.* **26**, 1006–1013. (doi:10.1177/0956797615576533)). They tested five listeners and found that perception (target detection accuracy) fluctuated rhythmically just like the sound rhythm. Here, we replicate the original finding, assess how likely the finding is to be observed for any individual, and quantify effect size in a large sample of adult listeners (*n* = 149). We introduce a model-based analysis approach that allows separate estimates of amplitude and phase information in target detection responses, and quantifies effect size for individual listeners. Together our results strongly support the presence of oscillatory influences on target detection accuracy, as well as substantial variability in the magnitude of this effect across listeners.

## 1. Introduction

Rhythm is a prominent feature of many behaviourally relevant stimuli. Music might spring to mind most easily, but other sounds (such as speech and animal vocalizations) and even the movements we generate with our own bodies (such as walking and chewing) are characterized by temporal regularities. In turn, rhythmic structure in the world around us is hypothesized to guide our attention to temporally expected future time points, allowing us to perceive and subsequently remember temporally expected events better than unexpected events.

Dynamic fluctuations of attention are hypothesized to be underpinned by synchronization of brain rhythms to stimulus rhythms (often referred to as *entrainment* [1]). Brain rhythms, or *neural oscillations*, reflect fluctuations of neuronal excitability and as such govern the likelihood with which a neuronal population will respond to a stimulus at any given time [2]. In turn, transient brain responses like event-related potentials (ERPs) and neuronal spiking differentiate stimuli that are rhythmically expected versus unexpected [3,4], confirming processing differences at the neural level. Neural oscillations synchronized (entrained) by a stimulus rhythm are thus a likely candidate mechanism for temporal attending [5,6].

Successful neural entrainment to speech rhythm is associated with better speech intelligibility [7], better memory for what was said [8] and better separation of the speaker’s voice from background noise [9]. Moreover, individual differences in auditory rhythm processing have been proposed to contribute to difficulty processing language [10,11]. Thus, the current scientific consensus can be summarized as follows: better neural entrainment underpins more precise temporal attending, which in turn leads to more successful perception of rhythmic stimuli.

However, neural entrainment and its behavioural consequences have recently been the target of existential doubts. For example, a special issue in the *European Journal of Neuroscience*, explicitly inviting null results and failures-to-replicate, is dedicated entirely to revisiting whether there is clear evidence for ‘rhythms in cognition’ [12]. The important theoretical questions that arise from this debate can be stated on the population as well as the individual level as follows. (i) Are behavioural benefits for rhythmically expected stimuli due to neural entrainment at all, or can they be explained by alternative neural or perceptual mechanisms? (ii) Is everyone susceptible to rhythmic entrainment? Following from this, (iii) what is the population distribution of behavioural effect sizes that can be expected based on a rhythmic manipulation?

Why is answering these questions so important? Rhythm, because of its assumed ability to entrain brain activity, perception and behaviour, is being increasingly used in therapeutic contexts. Rhythmic auditory stimulation is used widely in rehabilitation for stroke, Parkinson’s disease and traumatic brain injury [13]. Companies and startups are investing in software that supports flexible and interactive forms of rhythmic auditory stimulation [14]. Moreover, rhythmic non-invasive electrical brain stimulation entrains neural oscillations and is seen as a potentially promising on-the-horizon intervention that might improve perception for some individuals [15]. However, the efficacy of all of these rhythm-based therapies has been questioned, largely because there are substantial individual differences in responsiveness to these techniques [16–18]. Thus, it is critical to understand the magnitude of the influence of rhythm on perception in individual listeners. This is at least in part because the fundamentally clinical directions that rhythm research is currently headed depend on an honest assessment of the presence and stability of rhythm’s influence on behaviour.

**Figure 1.**
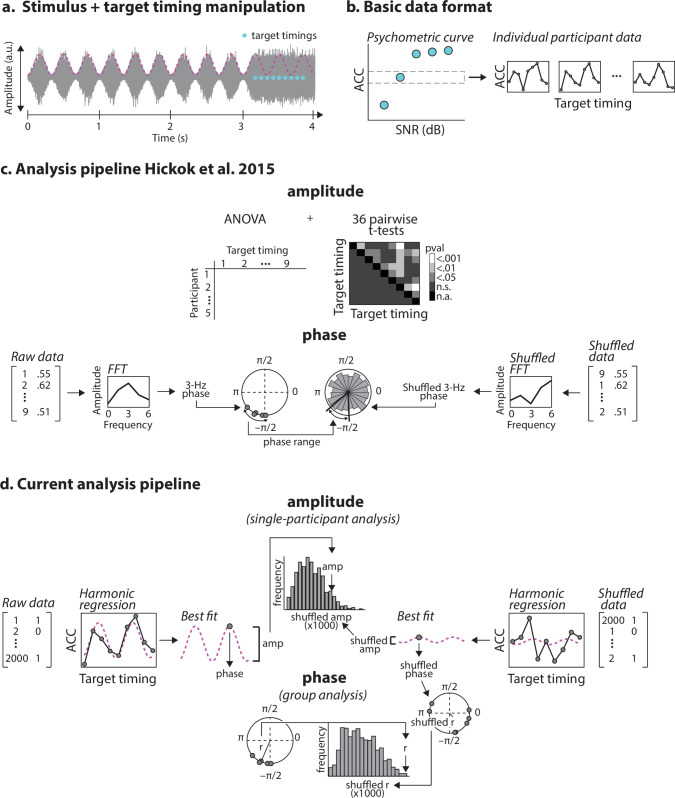
Auditory stimulus design and analysis pipelines. (a) Stimulus and target timing manipulation. Stimuli were 4 s white noises; the first 3 s were amplitude modulated at 3 Hz, and the final second unmodulated. On signal-present trials, a tone was presented at one of nine possible target timings (blue circles) in the unmodulated portion of the stimulus, and signal detection performance was analysed relative to the extrapolated 3 Hz rhythm (purple dashed line). (b) Basic data format. Target tones were presented at one of five signal-to-noise ratios (SNRs); the psychometric curve (left) shows accuracy as a function of SNR. Only the data from one SNR condition were analysed further (box). Individual participant data (right) were then analysed as a function of target timing. (c) Analysis pipeline of Hickok *et al*. [19]. Analysis of the amplitude of accuracy fluctuations (top) involves conducting a one-way repeated-measures on accuracy data, and then 36 pairwise *t*-tests (uncorrected for multiple comparisons) on all possible target-timing pairs. For analysis of the phase consistency of behavioural modulation 3 Hz phase was estimated from an FFT applied to each participant’s data. Accuracy data were then shuffled relative to their corresponding target-timing condition labels 1000 times per participant, and 3 Hz phase estimated for the shuffled data. The range of the empirical phase distribution was then compared to the range of the 5000-point surrogate distribution. (d) Current analysis pipeline. Accuracy values were predicted from target timings using harmonic regression, which yields amplitude and phase parameters for the sinusoidal best fit. Surrogate data were created by shuffling single-trial accuracy values with respect to target-timing condition labels 1000 times per participant, and surrogate data were submitted to harmonic regression. Single-participant *Z*-values were calculated for empirical amplitude values relative to the distribution of shuffled amplitude values. Phase clustering, indexed by resultant vector length, was then compared to shuffled resultant vector lengths.

Hickok *et al*. [19] introduced a clever behavioural paradigm with which to study how rhythmic context influences auditory perception. They presented listeners with a fluctuating sound (amplitude-modulated noise), followed by a steady-state sound, during which time a target tone might be present. Participants were tasked with indicating whether a tone was present or not. By varying the time at which the tone was presented, the authors were able to determine whether the timing of the tone—relative to the rhythm of the preceding amplitude-modulated noise—was related to whether it was perceived. Theoretically, one of the tell-tale signs of entrainment would be to observe oscillation,^1^ either neural or behavioural, continuing on *after* the cessation of a stimulus rhythm [21]. Hickok *et al*.’s study is so important because it shows *exactly this*—fluctuating auditory thresholds in the wake of an auditory rhythm—a hallmark of neural entrainment (figure 1).

However, uncertainty about the size of the effect in Hickok *et al*. [19] comes from at least three sources. First, no clear effect size was reported in the study. Second, the study involved a small sample size (five participants), making it difficult to generalize to a larger population [22]. Third, a follow-up study [23] re-analysed the data from the original paper instead of providing a replication, preventing comparisons between two independent samples. In addition to these sources of uncertainty, a recently published study by Sun *et al*. [24] failed to replicate the observation of behavioural oscillation at the group level and suggested that the effect is only present at the individual level in approximately one-third of listeners. However, as noted by Saberi & Hickok [25], Sun *et al*.’s replication was not, in fact, *direct*, as several experimental details differed from the original study. Thus, the degree to which the original finding is replicable by researchers not involved in the original report remains unclear.

Here, we replicate and expand the analytic approach employed by Hickok *et al*. [19] to quantify the degree to which individual listener’s behaviours are affected by rhythmic context. Importantly, we use harmonic regression to provide estimates of effect size for behavioural consequences of stimulus rhythm for each participant. In contrast to the ANOVA-based analysis framework of the original study (which is agnostic regarding the presence of *oscillation* in the data), our regression approach directly tests for oscillation-based fluctuations in listeners’ responses. In addition to applying our novel analysis pipeline, we increase the original sample size by more than an order of magnitude in order to provide a clearer sense of population variability in the effect size of behavioural oscillation in the wake of a stimulus rhythm.

## 2. Methods

### 2.1. Preregistration

This article received in-principle acceptance (IPA) at Royal Society Open Science. Following IPA, the accepted Stage 1 version of the manuscript, not including results and discussion, was preregistered on the OSF (https://osf.io/vygwk). This preregistration was performed prior to data collection and analysis.

### 2.2. Ethics information

Research was conducted under protocols approved by the institutional review boards of the participating researchers (certified by all analysis teams; see §2.3.1). Written informed consent was obtained from all participants. Participants were compensated monetarily or with course credit for their participation.

### 2.3. Design

#### 2.3.1. Multilab participation

Following acceptance of the Stage 1 report, additional labs were invited to take part in the study. Labs were recruited using social media and word of mouth (i.e. emailing colleagues in auditory science). All participating labs collected data using the same PsychoPy [26] script and written instructions (translated from English as needed). Participating labs self-identified as being equipped to conduct auditory psychophysics experiments, and used their own native hardware (soundcards, headphones); we collected information about each team’s auditory setup (see electronic supplementary material: Audio setup questionnaire for individual labs). Participating teams were required to contribute a minimum of five complete datasets (the sample size of the original report) for inclusion. Up to three researchers from each team were eligible for paper authorship based on data collection and contribution and reviewing the final manuscript.

#### 2.3.2. Stimuli and procedure

Stimuli were identical to those used by Hickok *et al*. [19].^2^ Stimuli were 4 s white noises, amplitude modulated for the first 3 s at a rate of 3 Hz, i.e. for 9 cycles, always beginning at the trough of the modulation (sin phase = 0), and a depth of 80% (figure 1a). The final second of the stimulus was unmodulated. Within the final unmodulated segment of the stimulus, the probability that a target tone was present in a trial was 50%. When present, the target tone occurred at 1 of 9 temporal locations relative to the 3 Hz rhythm, had it continued (+0, 0.25, 0.50, 0.75, 1.00, 1.25, 1.50, 1.75 and 2.00 cycles relative to the first expected amplitude peak; figure 1a). Target tones were 50 ms, 1000 Hz pure tones with 5 ms linear onset/offset ramps. Target tones were presented at 1 of 5 signal-to-noise ratios (SNRs) relative to the unmodulated noise segment (−18, −14.5, −12, −8.5 and −6 dB).^3^ The level of the unmodulated noise segment was calibrated to 70 dB sound pressure level (SPL) as in the original report; we provided a separate PsychoPy script for calibration. Sounds were presented over headphones.

For the registered replication, we opted to take a few minor liberties with the pseudo-randomization algorithm and experimental aesthetics relative to that employed by Hickok *et al*. [19], as described in the electronic supplementary material (table S2). First, Hickok *et al*. randomly chose stimulus information (target present versus absent, SNR and target timing) on each trial. Thus, they did not necessarily present equal numbers of trials across all conditions, and there were no restrictions on the number of similar trials that could occur successively. We chose to balance the number of trials presented per condition and per session, and additionally employed the pseudo-randomization restriction that no single stimulus condition (target present versus absent, SNR, target timing) could be presented on more than three consecutive trials. Each participant completed the experiment over the course of five sessions, during which they completed a total of 2250 trials. During each session, participants completed 450 trials (five observations per unique combination of target present versus absent, SNR and target timing; see electronic supplementary material, table S2).

Second, Hickok *et al*.’s experiment was performed directly in the Matlab command window, and participants were able to respond as soon as the trial began; that is, participants were not required to wait until the target had been presented (or not) before responding. We implemented a version of the experiment that restricts what the participant is able to see (limited to simple instructions, cues to stimulus-presentation time and prompts to respond *after* the stimulus has finished playing). Our experiment, programmed in PsychoPy version 2021.2.3 [26], was distributed to all participating labs to ensure consistency of instructions, stimulus delivery, and response collection.

At the end of each session, participants completed a brief post-experiment survey regarding their understanding of the task, their ability to maintain attention to the task, the effort they exerted and any strategies they may have used (see electronic supplementary material: Post-experiment survey). At the end of the fifth session, participants were asked to provide basic demographic information (e.g. age, sex) and complete a measure of self-reported hearing ability, namely the 15-item Speech, Spatial and Qualities of Hearing scale (15iSSQ) [27], as well as an assessment of musical abilities and training, namely the Goldsmiths Musical Sophistication Index (MSI) [28]. We used PsychoPy to administer the surveys.

### 2.4. Sampling plan

We used a sequential-*n* design using Bayes factor design analysis (BFDA) to help ensure that we collected sufficient evidence while maintaining efficiency in our design [29,30]. As an overview, in sequential designs, sampling is continued until the desired level of the strength of evidence is reached (i.e. Bayes factor; BF_10_), which in our case is 30 times in favour of the experimental hypothesis over the null hypothesis, or vice versa. In practice, we are confident that overall large effect sizes render this point somewhat less critical: The BF_10_ for our combined pilot data (*n* = 12) using a two-tailed Bayesian *t*‐test in JASP (version 0.14.1) is 143.9 (very strong evidence for an effect of rhythmic context).

We were more interested in providing a reasonable range of performance variability. As such, we decided to collect data from a minimum of 50 participants (an order of magnitude greater than the original study). In the unlikely event that we did not have a Bayes Factor of at least 30, we committed to continue to collect data, reaching at least 100 participants before discontinuing data collection. Note that sequential testing does not increase the risk of type I errors in a Bayesian framework [31].

Hickok *et al*. [19] reported testing five human adults with normal hearing, but did not provide any further information on inclusion or exclusion criteria. We screened participants for normal hearing abilities (self-reported). Moreover, we planned to test adults between the ages of 18−35 years, since both signal-detection in noise [32,33] and neural entrainment [34,35] have been shown to change with age. Nonetheless, we anticipate that this paradigm will afford directly investigating aging in future projects. Participants were excluded if the experimenter reported inattention or if participants self-reported that they did not understand the task in a post-experiment survey (see electronic supplementary material: Post-experiment survey). Finally, we decided to discard data for which participants did not achieve an accuracy of at least 0.8 for the easiest SNR (−6 dB condition).^4^

### 2.5. Analysis plan

Our primary research questions, hypotheses and the statistical tests we planned are summarized in table 1. In addition to testing whether we replicate the *presence* of rhythmic behavioural modulation in a larger sample than the original publication, a major goal of this registered replication was to provide information on the *distribution* and *variability* of the effect size in the population of young–adult listeners that can inform future investigations of the factors that would be expected to influence rhythmic behavioural modulation.

**Table 1.** Design table.

question	hypothesis	sampling plan (e.g. power analysis)	analysis plan	interpretation given to different outcomes
Is behaviour modulated by the stimulus rhythm?	H1: Yes. Given our pilot experiment, we expect to replicate the primary finding from the original paper, namely the presence of rhythmic behavioural modulation. H2: No.	see §2.4.	Z_A_ effect sizes will be tested against 0 using a single-sample *t*‐test	H1: We cautiously interpret, similarly to the authors of the original paper, that neural oscillations were entrained and continued after the cessation of the stimulus rhythm. However, we look forward to systematically investigating the mechanism underlying this phenomenon. H2: Given our pilot experiment, a failure to replicate flags that one of our changes to the protocol (electronic supplementary material, table S2) may have been critical. In this case, we will systematically investigate the small differences between our study and the original to see which of them mattered for the presence of the effect.
If yes, is the timing of behavioural modulation consistent across participants?	H1: Yes. Given our pilot experiment, we expect significant phase clustering of behavioural modulation functions across participants. H2: No.	see §2.4.	conversion of Z_r_ vector-length effect size to a *p*‐value (*normcdf*)	H1: We cautiously interpret, similar to the authors of the original paper, that neural oscillations were entrained with a similar phase lag for each participant. We look forward to systematically investigating the acoustic factors that may play a role in the degree of phase clustering across participants. H2: Given our pilot experiment, a failure to replicate flags that one of our changes to the protocol (electronic supplementary material, table S2) may have been critical. In this case, we will systematically investigate the small differences between our study and the original to see which of them mattered for phase clustering across participants.
Does behavioural-modulation effect size vary across sites (control)?	H1: No. We do not expect behavioural-modulation effect sizes to vary with site. H2: Yes.	see §2.4.	linear mixed-effects model with site included as a factor	H1: Experimental procedures were sufficiently stable across sites. H2: Site differences may flag variations in experimental setup or protocol. Our goal is to minimize this possibility by providing all sites with a standardized experiment, instructions and stimuli, but site differences will trigger an investigation into equipment and behaviours in any sites that differ from the larger dataset.

#### 2.5.1. Estimating single-participant effect-sizes

We had two goals while developing our analysis strategy. First, we wanted to distill the relevant dependent measures describing a behavioural *oscillation*. This is critical because an analysis strategy relying on ANOVA is not sensitive to whether the data possess rhythmic structure,^5^ whereas the underlying theory being tested mandates a quasi-sinusoidal behavioural oscillation. An additional benefit was avoiding 36 pairwise *t*-tests and the corresponding need to correct for multiple comparisons. Second, we wanted to offer a single-participant effect size measure (i) so that we could attempt to approximate population effect size in a larger study, and (ii) that could be used as a correlate for performance on independent tasks assessing, for example, speech comprehension or efficacy of an intervention on a single-participant basis. Here, we focus on data from the −14.5 dB SNR condition, as in the original publication.

For each participant, we performed a harmonic regression [36] where we predicted proportions of correct responses from target timing. Target timings, *T*, are transformed to phase values by multiplying them by 2π*f*, where *f* corresponds to the modulation frequency of the stimulus in Hz, *f* = 3. The conversion of target timings to phase values is necessary to quantify the strength of behavioural oscillation. Then, phase values are linearized for the regression by taking their sine and cosine, *x*_1_ = sin(2π*fT*) and *x*_2_ = cos(2π*fT*). In the end, for each participant, nine proportion correct values will be predicted from an intercept and the sine and cosine of the phase-converted target timings by solving the following equation:


$$y\;=\;\beta_{0}\;+\beta_{sin}x_{1}\;+\;\beta_{cos}x_{2},$$

using least-squares minimization. The resulting values of $\beta_{sin}$ and $\beta_{cos}$ will then be recombined to yield estimates of the amplitude


$$A=\sqrt{(}\beta_{sin}^{2}+\beta_{cos}^{2})$$

and phase


$$\phi =atan2\left(\beta_{sin},\beta_{cos}\right)$$

of the behavioural modulation. The amplitude parameters can be interpreted as a measure of effect size (in standardized units). Note that this harmonic regression approach is logically identical to fitting proportion correct data with a sine/cosine function and taking the resulting best-fitting amplitude and phase parameters, but is computationally much faster, which is a significant advantage for the permutation strategy that we use to estimate effect size (described below).

Amplitude parameters, *A*, are magnitudes and cannot be negative. Thus, estimates of *A* cannot be meaningfully tested against 0 to indicate the *presence* of a behavioural oscillation. Moreover, the amplitude of a behavioural oscillation may be compressed when performance is near ceiling or floor, not necessarily due to weaker entrainment, but rather to a compression of the range in which behaviour can fluctuate. To solve both of these problems, we employed a permutation strategy where single-participant null-hypothesis distributions are generated from each participant’s actual data. For each participant, on each of 1000 permutations, we shuffled single-trial binary accuracy values with respect to their corresponding target-timing condition labels (figure 1d). We then recalculated proportion correct for each of the 9 target timings and apply the same regression analysis described above. We form a ‘permutation distribution’ of amplitude parameters, *A*_p_, estimated from each of the 1000 regressions on shuffled data. Then, we compared the true amplitude parameter estimated from the original data to the permutation distribution by calculating a ‘robust *z*-score’,^6^ based on median absolute deviation (MAD [37]):


$$Z_{A}=\;\frac{0.6745\left(A-{\tilde{A}}_{p}\right)}{{MAD}_{A}}$$

where


$${MAD}_{A}=median\left(\left|A_{p}-{\tilde{A}}_{p}\right|\right)$$

and where the ~(tilde) symbol denotes the median.

*Z*_A_ values for each participant can be meaningfully tested against 0 to confirm presence of quasi-sinusoidal behavioural modulation, and moreover, act as a single-participant effect-size measure that is normalized by the participant’s own data and preserves descriptive statistics such as overall performance level, as well as individual-differences factors such as response bias.

Consistency of the phase*,* ϕ*,* of behavioural modulation across participants is also an important dependent measure, and clarifies whether the temporal structure of the behavioural modulation with respect to the stimulus rhythm was consistent across participants. Phase consistency of the ϕ parameters estimated from single-participant regressions can be quantified by the resultant vector length, *r*, of the sample of angles. Here, the dependent measure, *r*, is calculated across participants. Using our permutation strategy, we calculate a phase-lag value, ϕ_p_, for the behavioural modulation based on shuffled data. On each permutation, we then calculated resultant vector length, *r*_p_, across participants. The end result is a single, across-participants permutation distribution of *r*_p_ values against which the empirical r value can be compared


$$Z_{r}=\frac{0.6745\left(r-{\tilde{r}}_{p}\right)}{MAD_{r}},$$

where


$${MAD}_{r}=median\left(\left|r_{p}-{\tilde{r}}_{p}\right|\right).$$

The resulting *Z*_r_ value constitutes an across-participants effect size for phase clustering, and significance was tested by converting to a *p*-value using the cumulative normal distribution function, *normcdf*.

In the §2.6, we apply our proposed analysis pipeline to a small pilot dataset as a proof of principle.

### 2.6. Pilot data

As a first step and prior to acceptance of the stage 1 registered replication, we conducted a pilot experiment in which we collected data using the identical experimental and stimulus generation code as the original paper. Hickok *et al.* were kind enough to share their raw data and experimental scripts with us. We used their experimental scripts to replicate their study in a small sample (*n* = 7) in our own labs. Here, we briefly describe our own version of the study, and report our own data together with our reanalysis of the original data. The pilot experiment allowed us to tune the analysis pipeline that we proposed to use in this registered replication.

#### 2.6.1. Ethics information

For the pilot data presented here, data collection at the Max Planck Institute for Empirical Aesthetics (*n* = 2) was approved by the Max Planck Society Ethics Council. Data collection at Washington University (*n* = 5) was approved by the Washington University in Saint Louis institutional review board. Written informed consent was obtained from all participants.

#### 2.6.2. Stimuli and procedure

Stimuli were identical to those used by Hickok *et al*. [19] and are described in detail in §2.3.2. Stimulus generation, stimulus presentation and data collection were controlled by a custom Matlab script, written and provided to us by Hickok *et al*. On each trial, a single stimulus was presented; all condition info (target present versus target absent, SNR, target timing) was randomly selected on each trial. Participants responded whether each stimulus contained a target tone or not by pressing one of two keys on the computer keyboard; the response prompt was displayed and participants were able to enter their responses any time during the stimulus presentation. Corrective feedback was provided on each trial. Each block comprised 100 trials, and each participant completed 20 blocks (one participant completed 21 blocks) over the course of 5−8 sessions.

#### 2.6.3. Data analysis

##### 2.6.3.1. Direct replication

**Figure 2.**
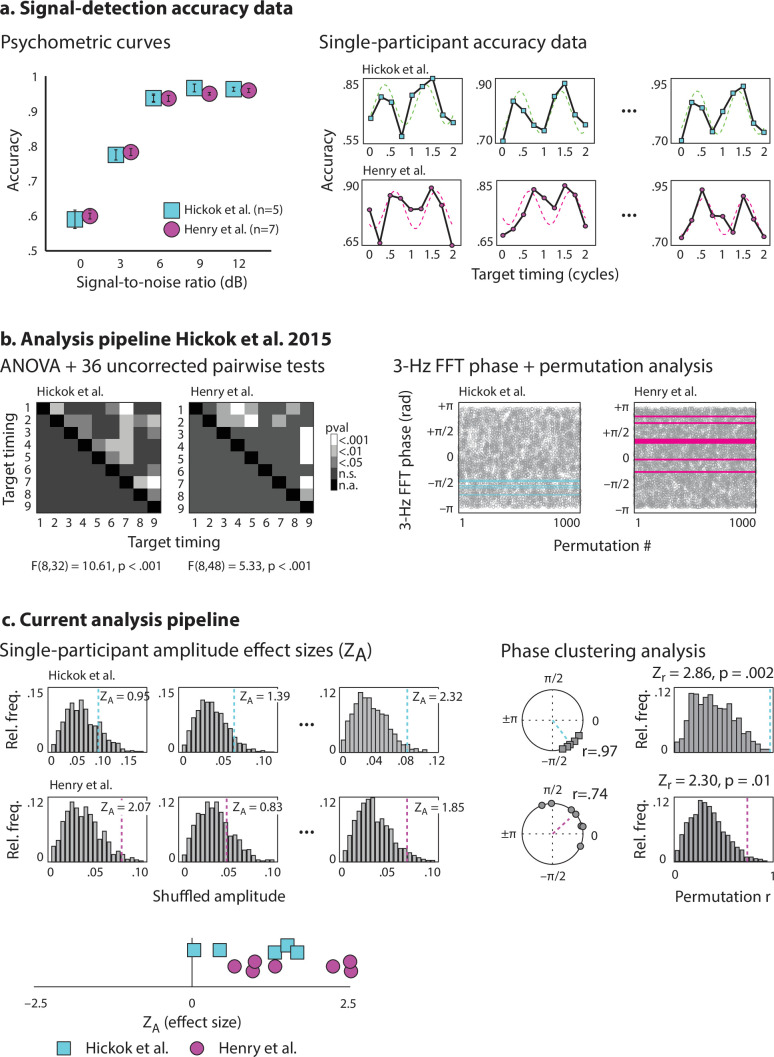
Data analyses using the pipeline reported by Hickok *et al*. and our proposed pipeline*.* (a) Signal-detection accuracy data. Left: psychometric curves plotting accuracy (proportion correct) as a function of target SNR for the data of Hickok *et al*. (blue squares) and our pilot data (magenta circles). Error bars show standard error of the mean. Right: individual participant accuracy data from the −14.5 dB SNR condition plotted as a function of target timing. Dashed lines show best-fit harmonic regression lines. Individual participant accuracy data for all participants are provided in electronic supplementary material, figure S1. (b) Analysis pipeline of Hickok *et al*. [19]. Left: both datasets were submitted to separate one-way ANOVAs with target timing as the sole within-participant variable. ANOVAs were significant for both datasets. ANOVAs were followed up by 36 *t*-tests contrasting accuracy for each pairwise combination of target-timing conditions; the *t*-tests were not corrected for multiple comparisons. In the dataset of Hickok *et al*., 20/36 *t*-tests were significant, and in our pilot data, 13/36 *t*-tests were significant. Right: phase clustering was tested for significance using a permutation-based approach. Per participant on each of 1000 iterations, the 9 accuracy values were shuffled relative to their target-timing labels, and 3 Hz phase was estimated from a FFT. The plots show empirical phase values for each participant (blue or magenta lines) plotted together with phases from shuffled datasets (grey circles) on each permutation (plotted on the *x*-axis). (c) Updated analysis pipeline. Left: single-participant estimates of amplitude effect size based on a permutation approach. Histograms show distributions of amplitude parameters estimated from data shuffled on a single-trial basis, and vertical lines show the amplitude estimate from the empirical data. Right: group-level phase clustering analysis for the dataset from Hickok *et al*. (above, blue) and our pilot dataset (below, magenta). Circle plots show empirical phase distributions with corresponding resultant vectors, and histograms show the distribution of resultant vector lengths obtained from 1000 permutations of the data on the single-trial level. Bottom: single-participant amplitude effect sizes combined across the two datasets.

First, we analysed our data exactly as described in Hickok *et al*. [19]. We calculated proportion correct (tone-detection hit rates and correct rejections) for each SNR.^7^ Next, we considered only the data for the −14.5 dB SNR condition, and calculated proportion correct separately for each target-timing condition. Examples of these single-participant performance curves are plotted in figure 2a. Following Hickok *et al*. [19], we conducted a repeated-measures ANOVA followed by pairwise *t*-tests between each pair of target timings (36 total tests, uncorrected for multiple comparisons). Finally, we scaled each single-participant proportion correct function between −1 and 1, and conducted a fast Fourier transform (FFT) on each. The 3 Hz phase angles from the FFT are plotted in figure 2b together with phase angles from surrogate data obtained by shuffling the nine proportion correct values relative to their target-timing condition labels and recalculating the FFT (1000 times for each participant). To statistically assess phase clustering, for the data of Hickok *et al*. [19], we calculated the proportion of iterations on which the shuffled phases all fell within –*π*/2 ± 0.5 rad.^8^ For our own data, we calculated the proportion of iterations on which the shuffled phases all fell within a phase range that we defined based on our own data, i.e. mean ± range/2, or *M* = 1.07 ± 1.39 rad.

##### 2.6.3.2. Estimation of single-participant effect sizes

We also applied our proposed analysis pipeline, which we describe in detail in §2.5.

##### 2.6.3.3. Demonstration of the pipelines on pilot data.

We applied the above-described pipelines (figure 1c,d) to our pilot data, as well as to the original data provided by Hickok *et al*. [19]. We present the results separately for the two samples as a ‘mini’ reliability check and preliminary replication. Figure 2a presents psychometric curves plotting accuracy (proportion correct) as a function of SNR for our pilot data and the data of Hickok *et al*. Moreover, we present examples of single-participant accuracy for the −14.5 dB condition with best-fit harmonic regression lines. Data for all individual participants are presented in electronic supplementary material, figure S1.

In order to recreate the analyses reported in Hickok *et al*. [19], we submitted both datasets to separate one-way ANOVAs with target timing as the within-participant variable. We reproduced the main effect reported by Hickok *et al*. [19] for their dataset (*F*_8,32_ = 10.61, *p* < 0.001) and moreover found a main effect in our own data (*F*_8,48_ = 5.33, *p* < 0.001). We followed up the significant main effects by conducting 36 *t*-tests per dataset contrasting each pairwise combination of target-timing conditions; we did not correct for multiple comparisons. Of these 36 tests, 20 were significant in the data of Hickok *et al*. and 13 were significant in our pilot dataset (figure 2b). We conducted FFTs on normalized accuracy data to quantify 3 Hz phase, and found significant phase clustering relative to shuffled data for the data of Hickok *et al*. (*p* = 0.004)^9^ as well as marginally significant phase clustering in our own data (*p* = 0.07).

In order to demonstrate our proposed analysis pipeline, we calculated single-participant amplitude effect sizes, *Z*_A_, for each participant based on distributions of amplitude parameters created for shuffled data.^10^ Figure 2c shows surrogate distributions together with empirical amplitude parameters for three example participants from Hickok *et al*. and three participants from our pilot dataset. Data for all participants are provided in electronic supplementary material, figure S1. We tested *Z*_A_-scores against 0, and found significant sinusoidal behavioural modulation in the data of Hickok *et al*. (*t*(4) = 3.46, *p* = 0.03) and in our own data (*t*(6) = 4.67, *p* = 0.003). Moreover, we combined the datasets so that we could make a first attempt at estimating a target effect size for sample size calculations for our registered replication (figure 2c, bottom). In the combined dataset, the mean amplitude effect size was *Z*_A_ = 1.51 (s.d. = 0.98), which was significantly different from 0 (*t*(11) = 5.37, *p* = 2.25 × 10^−4^). We also calculated phase-clustering effect sizes for the two samples, and found significant phase clustering for both datasets (Hickok *et al*.: *Z*_r_ = 2.86, *p* = 0.002; our pilot data: *Z*_r_ = 2.30, *p* = 0.01), as well as for the combined sample (*Z*_r_ = 2.36, *p* = 0.009).

In addition to testing for significant sinusoidal behavioural modulation within (amplitude) and across (phase-clustering) participants, our approach affords several advantages. These effect size measures can be used to test for (i) replication of the primary finding of sinusoidal behavioural modulation across experiments and sites, (ii) modulation by systematic manipulations to the acoustic or cognitive experimental situation (e.g. decreasing modulation depth or temporal regularity, changing the degree of ‘uncertainty’ by changing the number of signal-level conditions [23], or (iii) correlations with individual-participant trait or state variables (e.g. language abilities, musical expertise, etc.). Moreover, although we have in-principle replicated the original findings here with our pilot data, we used the stimulus generation and experiment delivery scripts provided by Hickok *et al*. Notably, a recently published failure to replicate [24] differed from the original publication in some of the same ways that we proposed to implement in our registered replication (electronic supplementary material, table S2). Thus, it was critical to show that the primary finding replicates when the design and randomization are improved, and as well to quantify the extent of individual differences in a large and geographically diverse sample of participants.

## 3. Results

Due to an oversight, our proposed upper age limit of 35 years was not clearly communicated to participating teams. Thus, the upper age of participants was 46 years rather than the proposed 35 years. There were no other deviations from the preregistered protocol for data collection or analysis.

### 3.1. Teams and participants

A total of 25 teams contributed participants; each team was randomly assigned a name of a cow breed to anonymize results.^11^ We had teams from 10 countries: Australia, Canada, Germany, Israel, Italy, the Netherlands, Russia, Switzerland, the United Kingdom and the United States. Each team certified that they received approval of their local ethics board and obtained permission for the sharing of deidentified participant data online.

Across all teams, we collected a total of 151 nominally usable participants (49 male, 102 female) aged 17−46 years at time of testing (*M* = 23.5, s.d. = 4.69). Teams contributed between 4 and 10 usable participants (median = 5).^12^ In line with our sampling plan, we conducted a one-sample *t*‐test of the Z_A_ values, which surpassed our criterion for stopping (BF_10_ = 3.235 × 10^17^).^13^

### 3.2. Post-experiment survey (exclusion criteria)

A brief post-experiment survey probing understanding, perceived difficulty and attention to the task was administered at the end of each session. We averaged ratings over the five sessions. Occasionally, participants did not provide ratings for some of the questions; 44 participants omitted at least one response during one session, but all participants provided ratings for all questions in at least three sessions (with the exception of one participant who omitted ratings in response to one question in three sessions), so we report average ratings over sessions where responses were provided. We preregistered an exclusion criterion that we would discard data for any participant that indicated that they did not understand the task based on subjective ratings (1 = ‘I understood very well what the task was’; 6 = ‘I did not understand the task’). Based on the distribution of responses to the question about task understanding, we chose to exclude two participants whose average rating exceeded 5. The remaining analyses thus include data for *n* = 149 participants.

**Figure 3.**
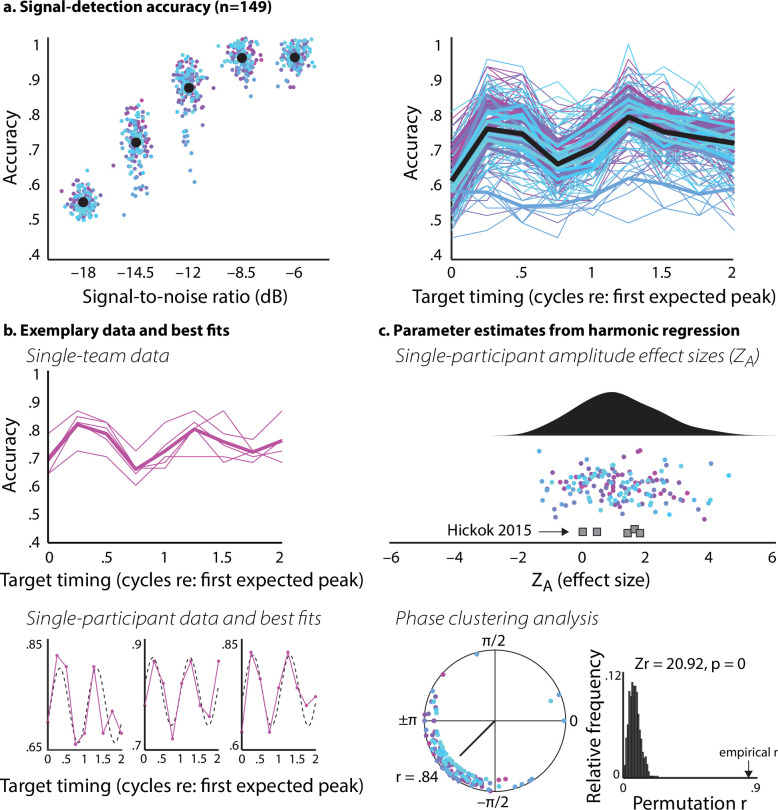
Accuracy data and parameter estimates from harmonic regression*.* (a) Signal-detection accuracy (*n* = 149)**.** Left: sychometric curves plotting accuracy (proportion correct) as a function of SNR. Each coloured dot is for a single participant, and each unique colour represents data from one team. Large black dots are the average over all participants. Right: accuracy as a function of target timing. Thin lines are single-participant data, again colored by team. Thick coloured lines are team means, and the thick black line is the average over all participants. (b) Exemplary data and best fits. Top: accuracy as a function of target timing plotted for a single team; thin lines are individual participants, thick line is team mean. Bottom: three example participants from the same team plotted above, with best-fit harmonic regression lines (dashed black lines) from which we estimated the parameters plotted in (c). (c) Parameter estimates from harmonic regression. Top: raincloud plot of Z effect size measures that quantify the amplitude of the sinusoidal modulation in the accuracy data, derived from the harmonic regression. Z effect sizes we estimated for the data from Hickok *et al*. [19] are shown below our data as grey squares. Bottom: the circle plot shows phase parameters from harmonic regression analyses and resultant vector (*r* = 0.84). Histogram shows *r*-values estimated from permuted data, and our empirical *r* is marked with a black arrow.

Psychometric curves plotting accuracy (proportion correct) as a function of SNR are shown in figure 3a. Consistent with the findings of Hickok *et al*. [19], accuracy for the most difficult (−18 dB) SNR condition was near chance and performance for the easiest two SNR conditions (−8.5 and −6 dB) was above 90%. No participant failed to achieve an accuracy of at least 80% for the easiest SNR (−6 dB condition), which was another preregistered inclusion criterion. Additionally, we also conducted all analyses with a subset of data adhering to a stricter cutoff—that is, on 109 remaining participants whose understanding-difficulty rating was not greater than 2. These results are reported in electronic supplementary material, figure S2 and are in line with the full analysis.

### 3.3. How strong is the rhythm of perception?

Next, we considered only the data for the −14.5 dB SNR condition and calculated proportion correct separately for each target-timing condition. These results are shown for the full sample in figure 3a, and examples of single-team and single-participant performance are plotted in figure 3b. We tested whether the mean of our *Z*_A_ effect size measures (*M* = 1.10, s.d. = 1.23) was significantly different from zero using a linear model predicting *Z*_A_ from an intercept term and team, where team was treated categorically. The intercept term was significant (t(124) =3.36, *p* = 0.001), confirming the *presence* of sinusoidal oscillation of accuracy as a function of target timing and critically *replicating* the original results reported in Hickok *et al*. [19]. Note that the *Z*_A_ values we estimated from the original paper’s data fall well within the distribution that we observe here (figure 3c).

Team also explained significant variance in our *Z*_A_ measures. The model including team significantly outperformed an intercept-only model (*F*_24,124_ = 1.96, *p* = 0.009). We will explore the causes and implications of site differences in the section below.

We also analysed the concentration of the phase parameters derived from the harmonic regression analysis using a permutation-based approach, whereby our empirical resultant vector length was compared against a distribution of resultant vectors that were calculated for shuffled data (§2.5.1.). Our empirical resultant vector length (*r* = 0.84) exceeded 100% of the values making up the permutation distribution (*Z*_r_ = 20.92, *p* < 0.001). Thus, the time course of the rhythm of perception was consistent across individuals (figure 3c).

### 3.4. Individual differences and survey responses

Although not part of our preregistered analysis plan, we collected information about individual differences (age, sex), as well as several survey measures for each participant (post-experiment survey [electronic supplemental material: post-experiment survey], 15iSSQ, Goldsmiths MSI). The distributions of responses or scores for all surveys are provided in electronic supplementary material, figures S3 (post-experiment survey), S4 (15iSSQ) and S5 (Goldsmiths MSI). As an exploratory analysis, we correlated age, post-experiment survey responses for each item, four scores calculated from the 15iSSQ (speech, spatial, hearing and composite), and the Goldsmiths MSI (general score) with our effect size measure (*Z*_A_). Correlation coefficients and corresponding *p*-values (uncorrected for multiple comparisons as this was exploratory) are provided in table 2. Only ratings of participant motivation were correlated with *Z*_A_, indicating that more motivated participants showed a stronger effect of the stimulus rhythm on target-detection performance.

**Table 2.** Correlations between effect size and individual difference measures.

measure	pearson *r*	*p* value
age	0.04	0.62
survey: understanding	−0.02	0.85
survey: difficulty	−0.08	0.35
survey: concentration strength	−0.11	0.19
survey: concentration change	−0.04	0.62
survey: concentration change direction	0.09	0.26
guessing	−0.13	0.13
motivation	0.23	0.005
SSQ: speech	−0.05	0.53
SSQ: spatial	−0.02	0.86
SSQ: qualities	−0.14	0.09
SSQ: composite	−0.10	0.24
MSI	0.07	0.41

### 3.5. Effects of team on conclusions about replicability

**Figure 4.**
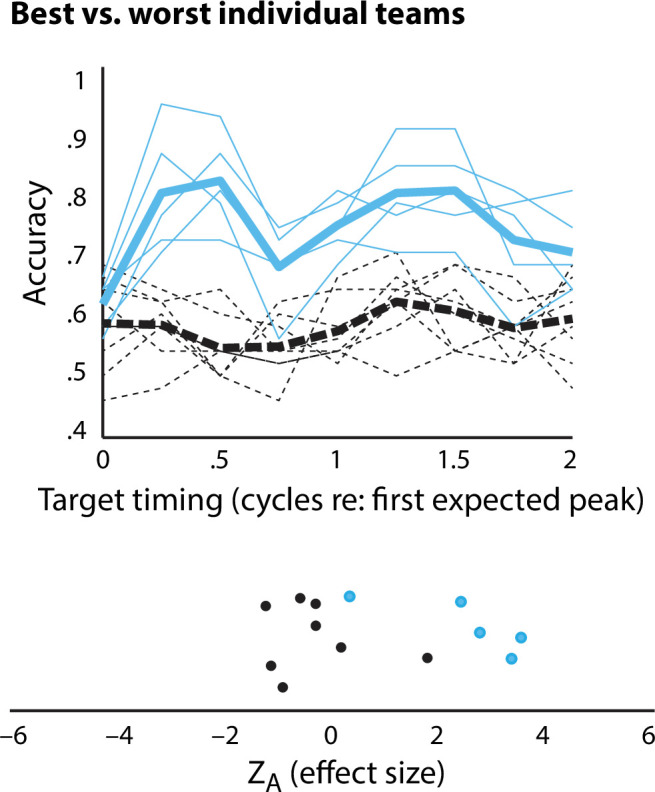
Illustration of variability in results as a function of team. Accuracy for the teams with the highest (‘best’, blue solid lines) and lowest (‘worst’, black dashed lines) average *Z*_A_ scores are shown. Thin lines indicate single-participant data, thick blue and thick black dashed line team averages over time. Top: accuracy as a function of target timing (cf. figure 3a). Bottom: *Z*_A_ scores based on our harmonic regression analysis (cf. figure 3c).

While inspecting the data, we noticed that there was large individual variability not just across participants, but also across team. Given the small sample size of the original publication, as well as subsequent questions about replicability, we decided to explore the magnitude of the effect of team. To illustrate the possible ‘random’ differences that may be present in any small dataset because of differences in audio setup, experimenter, etc., we compared the strength of the rhythm of perception for the best and worst team. For the teams with the largest and smallest *Z*_A_ scores, respectively, we plot their accuracy data and *Z*_A_ scores in figure 4. Although it is not necessarily meaningful to perform statistics after selecting for the strongest and weakest effects in a dataset, as an illustration, an independent-samples *t*‐test comparing *Z*_A_ scores for the two teams was statistically significant, *t*(11) = 4.54, *p* = 8.4195 × 10^−4^. Thus, if this replication had been conducted by any individual team, they may have ended up at opposite conclusions.

## 4. Discussion

In a large sample, we found robust evidence supporting that target detection accuracy is significantly influenced by the phase of preceding amplitude-modulated noise. This finding is consistent with listeners’ perceptual entrainment to rhythmic acoustic stimuli, and may relate to auditory entrainment in the context of other speech and non-speech contexts. We also uncovered a range of how individual listeners were affected by amplitude-modulated noise, which provocatively suggest individual differences in rhythmic auditory processing.

Our findings replicate and extend those of Hickok *et al*. [19]. Notably, our multi-lab replication exceeds the original sample size by a factor of nearly 30, facilitating examination of individual differences in behaviour. We also used a harmonic regression approach to better quantify the strength of entrainment in single participants.

Beyond testing the robustness of the primary finding, we also wanted to perform some initial analyses of individual differences in the effect size. As such, we assessed its relationship to age, and self-reported measures of task performance, communication success and music sophistication. These findings suggest that intersubject variability of the main effect is not easily explained by these metrics, and may instead point towards a fundamental ability (although we also note that, to our knowledge, psychometric properties such as test–retest reliability have not been assessed).

We found the process of the replication to be very useful in identifying possible reasons why some prior attempts to replicate this effect were not successful. Many of these related to specific aspects of the experimental design; for example, some researchers reported attempting to replicate the effect using only a single SNR (i.e. the critical SNR) [24], when in fact variability in task difficulty appears to be essential for achieving the effect [23]. This dependence on SNR variation is plausible in the context of physical considerations of true oscillatory entrainment, like the phenomenon of the Arnold tongue (i.e. an individual’s endogenous oscillator’s propensity for entrainment should depend on the exact match of the entraining frequency to its endogenous frequency, but also on the signal strength with which the entrainer is presented [38]).

Our results may also prove informative to discussions regarding the number of participants required for a scientific publication. Of course, there is a long history of small-*n* designs in some fields, including psychophysics, and these may continue to have their place [39]. However, our current results suggest several reasons small-*n* designs may be suboptimal—even in psychophysics. Notably, the variability of individual performance on our task (figure 4) means that any sample of five participants is unlikely to reflect the true effect size, and indeed some samples of this distribution would likely fail to detect a significant effect. In keeping with these observations, we found significant variation across testing sites (team was a significant predictor in the analysis), although this likely reflects a combination of individual differences in performance and idiosyncratic variations across testing sites.

Finally, we hope our multi-lab effort demonstrates how replication projects can also improve and extend original work. For example, we identified a number of details missing or unclear from the original study that we clarified (electronic supplementary material, table S1); we adopted a new harmonic regression analysis approach that we think better estimates amplitude and phase relationships of participants' behaviour; and we made all stimuli, presentation scripts, data and analysis code publicly available, which we hope will aid future exploration of this interesting phenomenon.

However, we also note that outstanding questions remain, including whether the effect is driven by auditory entrainment, attentional entrainment or some combination. Auditory entrainment relates to entrained oscillations generated locally in the auditory cortex. The phase of such oscillations could impact perceptual sensitivity [6,40]. We might expect auditory responses to be relatively insensitive to attentional manipulations, and to more strongly reflect acoustic properties of the stimuli (e.g. acoustic edges). Attentional entrainment refers to fluctuations in perception that originate beyond local sensory responses (i.e. top-down). Selective attention, temporal expectation, as well as stimulus difficulty are examples of such modulatory effects. Consistent with attentional entertainment, previous studies have suggested a role for variability in the overall distribution of difficulty across trials in the observed findings [23,24]. Our comprehensive multi-site report and model-based analysis approach provide powerful tools to move forward and better clarify the underlying mechanisms of the now robustly observed fluctuations in auditory performance.

## Data Availability

Data are available from [41]. Supplementary material is available online [42].

## Footnotes

^1^
A broader issue for the field is that it is also possible to have temporally structured attention that is not periodic [20], a possibility that becomes important when interpreting our results.

^2^
The original version of the script created stimuli in real-time for each trial. We modified the script to save stimuli to .wav files, which ensures the stimuli are consistent across sites and available for other researchers.

^3^
The original paper does not report the SNRs of the target tones relative to the masking noise. Instead, they report signal levels of the target tones relative to the quietest possible target tone (but see electronic supplementary material, table S1 for a note on the values they provide). In order to increase replicability of the methods, we diverge from the original manuscript here and report SNRs. This change is documented in electronic supplementary material, table S2.

^4^
The logic for this cutoff is as follows. We simulated random responding for the design we used in this registered replication (225 trials per each unique combination of signal present versus absent and SNR). For each of 100 000 random observers, we calculated accuracy for the easiest SNR. The highest accuracy we observed was 0.6. In our own combined pilot dataset, the mean accuracy for this SNR was 0.96, and even a liberal mean – (3 × s.d.) criterion would mandate a cutoff of 0.94. Thus, we chose a cutoff that approximates an average of those values (0.8). Our goal was to exclude participants who, for whatever reason, were unable to perform the task, while capturing the natural variability in performance.

^5^
In order to demonstrate this, we evaluated the false-positive rate of this ANOVA-based approach using the data from Hickok *et al*. [19]. On each of 1000 permutations, we shuffled each participant’s single-trial target-timing labels with respect to their binary accuracy data. Then we submitted each of the 1000 shuffled datasets to a repeated-measures ANOVA. Although the approach randomly paired condition labels and data, the ANOVA nonetheless reached significance 20% of the time with a nominal alpha-level of 0.05.

^6^
We have opted here for a robust *Z*-score based on the median and median absolute deviation (MAD) of the permutation distributions. This method is insensitive to the potential nonnormality of the permutation distributions, which may be problematic for a parametric *Z*-score based on mean and s.d.

^7^
The original paper states that analyses were conducted on the proportion of correctly detected tones; however, we followed the procedure as carried out in the analysis code provided by the authors, where noise trials were randomly assigned a SNR and a target-timing condition label, and proportion correct was calculated as the total number of hits (correctly detected tones) and correct rejections (correctly rejected noise-only trials), divided by the total number of trials.

^8^
The manuscript describes this procedure, whereby the shuffled phase distribution was compared to a range of –*π*/2 ± 0.5 rad. Our read is that these values seem to approximate the empirical mean and range of their phase values, *M* = –1.79 and range/2 = 0.47 rad.

^9^
Note that this analysis cannot be expected to replicate the precise *p*-value reported by Hickok *et al*. [19] (they report *p* < 0.0005), since the analysis is based on random data shuffling.

^10^
Since shuffling data on a single-trial level can disrupt temporal structure in a series of behavioural responses, we compared the *Z*_A_ values we obtained from our proposed pipeline to *Z*_A_ values calculated based on surrogate distributions that kept the behavioural time courses intact. Specifically, instead of randomly shuffling target-timing labels, we shifted the intact time course of condition labels relative to the intact time course of behavioural responses, so as not to destroy temporal structure in either time course. The *Z*_A_ values obtained from the two pipelines were correlated *r* = 0.9956 across the combined pilot sample (*n* = 12). Thus, we felt confident that the 3 Hz fluctuation we observed in the behavioural data was not an artifact of temporal structure in the behavioural time courses, and thus use condition-label shuffling to create surrogate distributions.

^11^
It is not entirely clear what prompted the cow connection. Informally we referred to this project as the multi-site overview of oscillations (MOO) which got the ball rolling, after which we decided to milk it for all it was worth.

^12^
The minimum number of contributed participants for inclusion in the project was five participants. However, after data were approved one participant was found not to have usable data. As it was a good faith attempt that team was not excluded from the project.

^13^
This analysis is for 149 participants, after excluding two based on self-reported ratings for understanding the task, as described in §3.
